# AI-mediated ultrasound radiomics in the diagnosis and treatment of triple-negative breast cancer: research progress and future challenges

**DOI:** 10.3389/fonc.2026.1802259

**Published:** 2026-04-30

**Authors:** Zhihe Wang, Yifan Wang, Tao Yu, Yan Yi, Ke Xue, Wei Xu

**Affiliations:** 1 Department of Ultrasonography, The First Affiliated Hospital of Yangtze University, Jingzhou, China; 2 School of Medicine, Hubei Polytechnic University, Huangshi, China; 3 Department of Medical Ultrasound, The Eighth Hospital of Wuhan, Wuhan, China; 4 Department of Ultrasonography, First People's Hospital of Wancheng District, Nanyang, China

**Keywords:** artificial intelligence, radiomics, translational medical research, triple negative breast cancer, ultrasonography

## Abstract

Triple-negative breast cancer (TNBC) is a subtype of breast cancer with strong invasiveness, limited treatment options, and poor prognosis. Its early accurate diagnosis and individualized treatment are major challenges faced by the clinic. With the rapid development of artificial intelligence (AI) technology, AI-mediated ultrasonic radiomics provides new ideas for non-invasive diagnosis and treatment of TNBC. This technology integrates machine learning (ML), deep learning (DL), and radiomics methods to achieve high-throughput extraction of quantitative features from ultrasonic images and construct a predictive model capable of characterizing tumor heterogeneity. Currently, AI-driven ultrasonic radiomics for TNBC diagnosis has evolved from basic differential diagnosis to multi-subtype classification, with its diagnostic performance further improved via multimodal image fusion. For prognostic assessment, the models effectively predict patients’ disease-free survival (DFS) and overall survival (OS) by integrating intratumoral and peritumoral texture features, clinicopathological indicators, and other relevant factors. Nevertheless, the translation of this technology into routine clinical practice faces multiple challenges: insufficient standardized data protocols, limited model interpretability, and lack of rigorous multicenter validation studies. In the future, research on the establishment of a standardized radiomics workflow among different devices and medical centers should be given priority as well as the research on the construction of high-performance AI models with good interpretability, and multicenter prospective clinical studies should be carried out to verify its clinical value.

## 1 Clinical features and current status of diagnosis and treatment of triple-negative breast cancer

Breast cancer is the most frequent non-skin malignant neoplasm in women worldwide. Its incidence and mortality are among the highest in the world and have become a major public health problem (1). TNBC is a highly invasive molecular subtype of breast cancer with the absence of estrogen receptors (ER) and progesterone receptors (PR) on the tumor cells as well as no amplification of human epidermal growth factor receptor 2 (HER2) gene or no protein over-expression (2, 3). TNBC accounts for approximately 15%–20% of all breast cancers. Because it is of unique molecular phenotype, TNBC is resistant to primary endocrine therapy and anti-HER2 targeted therap. It has limited systematic therapy options and thus poor prognosis (3, 4). TNBC patients’ survival at 4 years is just at 77.0% rather than 92.5% of HR+/HER2- Luminal A type patients (5).

The invasive clinical characteristics of TNBC are often shown when it is diagnosed. Compared with the non-TNBC group, the TNBC group has very great tumor volume, with an average diameter of approximately 3.0 cm, and the average diameter of the tumor on non-TNBC groups is approximately 2.1 cm. At same time, the lymph node metastasis rate is bigger (54.6%) compared with that of non-TNBC at 45.6%; indeed there is much difference between the two groups. The recurrence pattern of this subtype is also stereotyped. There will be a peak in the risk of distant recurrence within 3 years after diagnosis, and its 5-year distant recurrence rate is about 2.6 times that of non-TNBC patients (6).

For treatment, chemotherapy based on anthracylines plus taxanes is still the mainstay of therapy for the systemic treatment of TNBC because of the absence of distinct molecular targets (7). Neoadjuvant chemotherapy is the most valuable choice for TNBC patients, which aims to reduce the size of the tumor first and then see if the tumor responds to the chemotherapy or not. Achieving pathological complete remission is a good predictor of TNBC, which means that you are going to survive for a long time if you do it, and if you fail to achieve it, the patients will be at a risk of recurrence and have a bad outcome (8).

Though we are using immunohistochemical and fluorescence *in situ* hybridization examination as the gold standard for TNBC diagnosis, it is still a very difficult diagnosis clinically. Some TNBC have atypical pathological features, or the limited sensitivity and specificity of the detection techniques could result in a delayed diagnosis or no diagnosis at all (9). On imaging, some TNBC lesion areas might be hidden or appear as pseudonecrotic lesions during routine examinations such as for ultrasound and mammography. Magnetic resonance imaging has greater value for detecting and assessing such tumors due to its higher sensitivity (10), but its widespread use is limited by its being expensive and time-consuming. In addition, in clinical practice, for cases with HER2 status at the critical threshold, if FISH testing is not routinely conducted for confirmation, it may also affect the accuracy of TNBC typing (11).

The diagnosis and treatment of TNBC are hard because of its special phenotype, its being highly invasive, its limited treatment options, and its being associated with poor outcomes. Improving the integration of multimodal imaging and precision pathology in order to enhance early diagnosis, shorten time to diagnosis, and guide personalized, strengthened adjuvant therapy based on neoadjuvant therapy result is key to enhancing TNBC’s result.

## 2 Traditional radiological assessment system for TNBC

Clinical detection and evaluation of TNBC are done by using current multimodal imaging systems. Mammography, as the basic screening method, is irreplaceable in detecting characteristic microcalcification (12). On mammogram, TNBC is often seen as an ill-defined mass with an ill-defined margin, which is different from other subtypes and usually associated with highly invasive pathological types (13). Ultrasound is a radiation-free and real-time imaging modality with the ability to assess hemodynamic phenomenon, etc. (14). Therefore, the combination of X-ray and ultrasound has become a routine method for breast cancer screening, especially in women with dense breasts, and can improve the detection rate of lesions (15, 16). Studies have shown that the 3.0-T MRI shows a higher diagnostic ability for the detection of TNBC, with a diagnostic sensitivity and specificity of 82.56% and 76.19% respectively. Its sensitivity was 77.91%, and its specificity was 78.57%, both higher than those of mammography. Combining these two imaging modalities can further improve the diagnosis, and the combined diagnosis reached a sensitivity of 90.70% and a specificity of 86.36% (17). Therefore, the “X-ray + ultrasound + MRI” combined strategy is commonly used in clinical practice to compensate for the shortcomings of a single modality: X-ray is good at detecting calcification but cannot distinguish soft tissues. Ultrasound is easy to operate but depends on the experience of the doctor. The precision of MRI is high, but the cost is high and it takes a long time.

Meanwhile, the traditional imaging evaluation system has a deficiency. If the diameter of the TNBC lesion is less than 1 cm or there is an atypical manifestation in the image, even if you use multiple modalities technology, the accuracy of detecting and analyzing qualitatively will have a huge decrease (18). The basic cause of this limitation is that traditional diagnosis is highly dependent on the subjective visual interpretation of radiologists, making it hard to achieve a non-subjective and reproducible method of determining the rich information contained in the images (19).

## 3 Inherent limitations of ultrasound imaging and the compensatory role of AI in radiomics

Ultrasound, as a key part of multimodal breast imaging, has been broadly used in clinical settings given its real-time imaging capability, low cost, and absence of radiation exposure (20). It also shows reasonable resolving power for soft tissues—lesion margins, echo texture, and acoustic differences can all be captured—which gives radiomics a decent information base to work with (21). Still applying ultrasound in quantitative radiomics is not without its problems. The imaging is heavily operator-dependent, and differences in scanning technique or plane selection alone can create meaningful heterogeneity between images (22). Image quality issues like speckle noise and acoustic artifacts add another layer of variability (23), and limited penetration depth makes visualization of deeper lesions genuinely difficult (24). Taken together, these factors directly undermine how stable and reproducible radiomics features are when data comes from multiple centers (25).

Radiomics is fundamentally about pulling quantitative features from images to support clinical decisions, but the limitations cited above create real interference at the feature extraction stage (26). Deep learning can help here—automated preprocessing, speckle reduction, and feature harmonization can go some way toward evening out the operator-related inconsistencies (27). That said, there are physical boundaries AI simply cannot cross: if a lesion is obscured by acoustic shadowing or sits beyond useful penetration depth, no algorithm is going to reconstruct what was never captured in the first place (28). Thus, while AI does meaningfully improve feature stability, none of it works well without a standardized acquisition protocol as the starting point (29).

## 4 Evolution and application of the AI technology system in medical radiological image analysis

Artificial intelligence (AI) serves as the methodological basis for radiologic applications in healthcare. Driven by machine learning (ML) and deep learning (DL), AI is closely integrated with radiomics, jointly promoting the development of precision medicine (30).

AI in medical radiological analysis is based on the continuous development of basic theories and algorithms. ML, an approach to implementing AI, has a theoretical history dating back to the mid-20th century; Rosenblatt’s 1958 perceptron model laid the foundation for subsequent data-driven modeling (31). In 2012, AlexNet of Krizhevsky et al. achieved breakthrough results in the ImageNet Competition, marking the first application of DL in complex image recognition. Soon afterward, DL rapidly expanded into the field of medical radiology (32). To meet the clinical demand for high-throughput extraction of quantifiable information from medical radiological images, Lambin et al. formally proposed the concept of “radiomics” in 2012 (33). This approach aims to convert the pixels of an image into a flexible high-dimensional dataset, non-invasively decode the biological properties of tumors (e.g., heterogeneity) without interference, and systematically extract quantitative radiological features (34).

Although ML, DL, and radiomics all belong to the AI context and are often combined in use, it is necessary to clarify their hierarchical relationship as well as their primary functional roles. AI gives us a macro methodological view; ML is an approach to implementing AI that learns data patterns via algorithms to make predictions or decisions. DL is a subset of ML that utilizes deep neural networks to process medical images. Radiomics, as a specialized medical imaging technique, uses ML/DL algorithms to generate models from extracted quantitative features (35, 36). This integration provides a novel approach for the early differential diagnosis, molecular subtyping prediction, and treatment response evaluation of TNBC (37).

## 5 Application of AI technology in multimodal breast imaging analysis and diagnosis decision-making

AI technology has been fully integrated into breast imaging analysis, promoting the in-depth integration of ML, DL, and radiomics with multimodal images such as mammography, CT, MRI, and PET/CT and bringing a series of breakthroughs in the precision diagnosis and treatment of breast cancer (38).

In the field of mammography, DL algorithms represented by deep convolutional neural networks (DCNN) have achieved diagnostic performance in lesion detection and risk prediction that is close to the level of professional radiologists (39). In breast CT, DL-based automatic segmentation technology can achieve high-precision mass segmentation, and the quantitative image features generated by it exhibit comparable discriminative power to that of radiologists in distinguishing between benign and malignant tumor (40). For breast MRI, DL technology is driving progress in key areas such as lesion detection and classification, radiogenomic research, and prediction of neoadjuvant chemotherapy efficacy (41). At the molecular functional imaging level, AI-driven radiomics analysis has also been applied to the PET/CT evaluation of breast cancer. By analyzing PET/CT images, AI can extract quantitative features that go beyond traditional visual interpretation. These features have great potential in improving tumor staging accuracy, predicting prognosis, and decoding tumor biological characteristics (42).

## 6 Application and discussion of AI-driven ultrasonic radiomics in TNBC

### 6.1 Diagnostic scenarios

First of all, for the single task of improving diagnostic performance, many studies have focused on how to make the model better at distinguishing TNBC—for instance, Lee et al. developed an ultrasonic radiomics score using LASSO regression for feature dimensionality reduction and subsequently performed model construction to better differentiate TNBC from fibroadenomas (43). Ma et al. and Wu et al. conducted independent studies using a decision tree and conventional DL, respectively, to obtain highly accurate TNBC classification, achieving AUC values of 0.971 and 0.88, respectively (44, 45). The DL model constructed by Zhang et al. also achieved an AUC of 0.864 for this task (46).

Secondly, further research has focused on the more challenging multi-subtype fine classification and multimodal information fusion, aiming to reveal the in-depth correlations between image features and molecular biological characteristics. The DL model developed by Jiang et al. achieved extremely high accuracy in the overall classification of molecular subtypes (47). The multimodal attention convolutional neural network (ACNN) model constructed by Zhou et al. performed particularly well in distinguishing TNBC by integrating grayscale ultrasound, color Doppler, and elastography features. Its AUC ranges from 0.934 to 0.970, and its efficiency even surpasses that of core needle biopsy (48). Ferre et al. and Gong et al., respectively, validated the ability of their models in multi-subtype identification. Among them, the model of Ferre et al. can effectively distinguish TNBC from non-TNBC as well as HER2-positive and HER2-negative subtype (49), while the study of Gong et al. showed that introducing contrast-enhanced ultrasound can significantly enhance the ability to distinguish specific subtypes such as Luminal A (50).

Finally, a key trend of research will be to address the clinical side and also direct means to meet the clinical need to focus on clinical practicability of the models as well as interpreting those models and how we can make these models more clinically useful to improve clinical decision making—for example, for Zhang et al., the model AUC of 0.864 was achieved, and it was also quantified to have a clinical value, showing that the model could help 67.86% of the BI-RADS 4a patients avoid unnecessary biopsies (46).

The radiomics nomogram constructed by Xu et al. had an AUC of 0.813 and was confirmed to have the potential to become a novel biomarker (51). Cai et al. increased the AUC of the TNBC recognition model to 0.71 by integrating ultrasound radiomics with clinical variables and revealed a clinical association wherein TNBC is more common in BI-RADS category 4 lesions (52). In addition, Boulenger et al. analyzed the feature learning process of the model using t-SNE visualization technology, enhancing the transparency of the model’s decision-making (53). **Table 1** summarizes the ultrasound radiomics diagnostic technologies for TNBC.

**Table 1:** Performance comparison of AI-driven ultrasound radiomics in the diagnosis of triple-negative breast cancer.

Clinical Events	Test methods	Accuracy	Sensitivity	Specificity	AUC/C-index	Author (Publication Time)
Identify TNBC from benign lesions	Ultrasound Radiomics	——	——	——	0.910 (training set) and 0.853 (validation cohort) for iU22 subgroup.	Si Eun Lee et al 2018.8 (43)
Identify TNBC from non-TNBC	Mammography + Ultrasound + Decision Tree (DT)	0.947	0.905	0.941	0.971	Mengwei Ma 2021.12 (44)
Identify TNBC from non-TNBC	Conventional ultrasound (Gray-scale) + Color Doppler + Machine Learning (Logistic Regression)	——	——	——	0.850→0.880	Tong Wu et al., 2018.9 (45)
Molecular typing of breast cancer (TNBC vs Non-TNBC)	Ultrasound deep learning (Xception)	0.533	——	——	0.864	Xianyu Zhang et al., 2021.3 (46)
Identify TNBC from non-TNBC	Ultrasound deep learning (ACNN)	——	——	——	0.934–0.970	Bo-Yang Zhou et al.,2021 (48)
Identify TNBC from non-TNBC	Ultrasound radiomics with logistic regression	——	0.818	0.742	0.824	Romuald Ferre et al.,2023 (49)
Diagnosis of breast cancer	CUS + CEUS radiomics	0.854	——	——	0.953	Xuantong Gong et al.,2023.5 (50)
Identify TNBC from non-TNBC	Ultrasound deep learning (VGG)	0.850	0.860	0.860	0.860 (CL:0.64–0.95)	Alexandre Boulenger et al.,2022.12 (53)
Identify TNBC from non-TNBC	CUS+Radscore nomogram	——	——	——	0.813	Maolin Xu et al.,2023.10 (51)
Identify TNBC from non-TNBC	ultrasound radiomics (Elastic Net) combined with clinical variables	——	——	——	0.710	Lie Cai, et al., 2024.3 (52)

In summary, the field has gradually evolved from validating basic differentiation capabilities to constructing intelligent auxiliary systems for complex scenarios, integrating in-depth multimodal information fusion and incorporating clinical interpretability.

### 6.2 Prognostic assessment

Initial research has confirmed that it is possible to use ultrasonic radiomics features combined with clinical indicators for prognosis. Yu et al. (54) developed a nomogram using multicenter mixed data on intratumoral and peritumoral texture features within 4 mm. Subsequent studies have focused on improving the robustness and performance of models via algorithmic optimization and multimodal fusion strategies. Wang et al. (55) addressed data imbalance using the SMOTEENN algorithm and then constructed a combined model integrating radiomics and clinicopathological features. This model demonstrated good predictive capabilities for disease-free survival, with an AUC of 0.90—significantly higher than that of the single-modality model. Hu et al. (56) have also constructed a more refined prediction model by incorporating radiomic scores and other medical data. The model performed well in the validation sets, exhibiting good performance with an AUC of 0.847 in the validation sets. At the same time, the study confirmed that the model can be used for preoperative evaluation of the level of tumor-infiltrating lymphocytes and established a connection between the radiological phenotype and the tumor immune microenvironment. Cutting-edge research aims to achieve long-term survival prediction and develop an automated analysis pipeline. Wen et al. (57) developed a combined nomogram by integrating automatic segmentation technology and DL methods, which accurately predicts the 5-year all-cause mortality risk of non-metastatic TNBC patients. The model obtained an AUC of 0.93 for internal validation and 0.89 for external validation, and its excellent performance indicates that this predictive tool will achieve long-term and stable clinical application. **Table 2** summarizes the ultrasound radiomics prognostic prediction for TNBC.

**Table 2:** Comparative analysis of model performance in AI-driven ultrasound radiomics for the prognostic assessment of triple-negative breast cancer.

Clinical events	Test methods	Accuracy	Sensitivity	Specificity	AUC/C-index	Author (publication time)
Prediction of DFS in TNBC	Ultrasound radiomics with clinical nomogram	——	——	——	C-index: 0.75 (training cohort)	Feihong Yu et al., 2021.8 (54)
Predicting DFS in TNBC	Ultrasound radiomics + clinical features + SMOTEENN	——	81.8%	82.3%	AUC 0.90	Haoyu Wang et al., 2022.12 (55)
Predicting TILs in TNBC (high vs. low)	CUS + radiomics (Clin + RS)	——	——	——	AUC 0.847 (validation)	Ling Hu et al., 2024.4 (56)
Prognostic prediction of OS/DFS in TNBC	Ultrasound radiomics (YOLO V3 + PyRadiomics) + Clinicopathological + XGBoost	——	——	——	AUC: 0.73–0.93 (internal/external validation)	Wenwen et al., 2025 (57)

Although these have all been great strides forward, they still face several challenges. Current research has limitations in the biological interpretation of feature selection, the reliability of automatic segmentation, and the standardization of multicenter data (55, 56).

## 7 Discussion

The application of AI-mediated ultrasonic radiomics in the diagnosis and treatment of TNBC has both merits and challenges (52). Although this approach has shown promising results in molecular subtyping and prediction, it still needs to address three technical obstacles to become a routine clinical test in the future.

The main difficulty lies in the lack of standardized data collection and processing procedures. Most current studies are based on single-center, small-sample retrospective data. There are variations in ultrasonic equipment, acquisition protocols, and operating methods across different centers, leading to radiomics feature discrepancies and reducing the generalizability of the developed models (43, 58). There are discrepancies in peritumoral region annotation among different observers, and numerous distinct pipelines exist for radiomics feature extraction, while confounding factors such as postoperative treatment also affect the robustness and reproducibility of the predictive model (54).

Moreover, there remains a lack of interpretability and correlation with biological relevance. Although feature selection methods such as LASSO regression can effectively construct highly predictive models (59), most ML and DL algorithms still have a “black box” nature (60). There is no clear mechanism underlying how models utilize features, and the association between specific features and distinct TNBC biological behaviors remains unclear, such as which feature is linked to a specific gene expression or tumor microenvironment heterogeneity (61, 62).

Addressing this interpretability gap also requires deeper biological grounding of radiomics features. The emerging paradigm of radiogenomics seeks to bridge the macroscopic imaging phenotypes with underlying molecular mechanisms (63). Recent studies have shown that specific ultrasound radiomic features can indirectly reflect tumor gene expression profiles and microenvironmental states, such as TIL abundance and immune phenotype (64). This provides a non-invasive means of characterizing the tumor molecular landscape and predicting immunotherapy response, which is of particular relevance given the increasing role of immune checkpoint inhibitors in TNBC treatment (65). However, current evidence remains largely associative, and the mechanistic links between specific radiomic features and genomic alterations or immune regulatory networks remain to be established (66).

Finally, practical difficulties have been encountered during the clinical translation process. These problems are reflected in terms like small sample sizes and lack of prospective validation (67, 68) and the problem of different levels of acceptance by clinical doctors due to different views of this technology (69) as well as problems related to ethics and regulation such as privacy protection and fair use of algorithms (70).

To overcome these obstacles, future work will focus on the following three points: on the one hand, set up a standard radiomics analysis pipeline for different devices (22, 71, 72); on the other hand, create a new generation of AI models with powerful prediction ability and good interpretability and promote understanding of radiomics markers in the pathogenesis of tumor biology (73). Lastly, prove the clinical value and cost-effectiveness of the models in large-scale multicenter prospective studies to promote the transition from the technical confirmation stage to the clinical application stage (50, 74).

## 8 Summary and outlook

AI-mediated ultrasonic radiomics holds promise for precise diagnosis and treatment of TNBC noninvasively. It is achieved through the exploration of tumor radiomics heterogeneity, which improves differential diagnosis and prognosis stratification.

The field is trending from traditional feature engineering to DL, from single-modal to multimodal, and interpretability has become the main direction. There are obstacles to clinical translation: poor generalizability, limitations of single-center, small-sample studies, and barriers to application.

Future breakthroughs will require the establishment of a standardization system and encourage multicenter data sharing. Efforts should be devoted to developing accurate and interpretable AI models, exploring the integration of multimodal data and radiogenomic correlations, and developing supporting clinical and ethical governance systems.
